# Development of loop-mediated isothermal amplification (LAMP) assays for the detection of diarrheagenic *E. coli* in wastewater

**DOI:** 10.1128/aem.00880-25

**Published:** 2025-08-11

**Authors:** Meret Zimmermann, Markus Schuppler, Timothy R. Julian, Seju Kang

**Affiliations:** 1 Department of Environmental Microbiology, Eawag, Swiss Federal Institute of Aquatic Science and Technology 28499, Dübendorf, Switzerland; 2 Laboratory of Food Microbiology, Institute of Food, Nutrition and Health, Swiss Federal Institute of Technology Zürich 27219, Zürich, Switzerland; 3 Swiss Tropical and Public Health Institute 30247, Allschwil, Switzerland; 4 University of Basel 27209, Basel, Switzerland; Universidad de los Andes, Bogotá, Colombia

**Keywords:** loop-mediated isothermal amplification (LAMP), diarrheagenic *E. coli*, wastewater-based surveillance (WBS), lateral flow, point-of-care (POC)

## Abstract

Diarrheagenic *E. coli* is responsible for a substantial portion of foodborne diseases globally. The use of standard diagnostic tools for the detection of diarrheagenic *E. coli* often hampers the establishment of robust surveillance when no expensive laboratory equipment, such as thermocyclers, is present. Loop-mediated isothermal amplification (LAMP) has shown potential to enable the resource-efficient detection of pathogens. In this study, LAMP assays with two detection modes, fluorescence-based molecular beacon (MB) and nucleic acid lateral flow (NALF), were developed for the detection of the diarrheagenic *E. coli* strains Shiga toxin-producing *E. coli* (STEC), enteropathogenic *E. coli* (EPEC), and enterohemorrhagic *E. coli* (EHEC). Duplex LAMP assays for the virulence genes *eae* and *stx2* of STEC, EPEC, and EHEC were developed and validated against wastewater from an on-site containment as a model environmental medium for wastewater-based surveillance in non-sewered areas. The developed LAMP assays showed moderate specificity toward *eae* and *stx2*, enabling crude differentiation among STEC, EPEC, and EHEC. We assessed the sensitivity of the LAMP assays and estimated a Limit of Detection (LoD) of 10^2^–10^3^ gene copies per reaction and found moderate quantitative capability for the MB-based method. The development of LAMP assays for the specific detection of STEC, EPEC, and EHEC with two distinct detection modes provides various options for their surveillance in settings without access to thermocyclers.

**IMPORTANCE:**

Foodborne diarrheagenic *E. coli* poses a public health threat, while the variability in transmissible agents hampers outbreak investigation. The lack of lab equipment, such as thermocyclers, in some laboratory settings obstructs the establishment of robust diagnostic tools. This study addresses the need for reliable diagnostic tools for thermocycler-independent application. Validation against wastewater from an on-site containment demonstrates detection of the targets in an environmental matrix that could provide representative epidemiological insights.

## INTRODUCTION

Approximately 5%–10% of the global population suffers from infectious diseases caused by foodborne pathogens (1). The consequences of foodborne infections cause a yearly global estimate of more than 32 million disability-adjusted life years (DALYs), with diarrheagenic *Escherichia coli* (*E. coli*) being responsible for the largest share (2). Diarrheagenic *E. coli* is generally defined as strains with genes able to produce virulence factors (3). Some types of diarrheagenic *E. coli* have been associated with severe and life-threatening diseases (4–7). Enteropathogenic *E. coli* (EPEC) harbors the genomic Locus of Enterocyte Effacement (LEE) containing *eae* that encodes the adhesive factor intimin and colonizes the human intestine (3, 8). Shiga toxin-producing *E. coli* (STEC) produces colonic receptor-specific toxins (3) that inhibit the colonocytes’ ability to synthesize proteins until the affected cells become necrotic (9). Enterohemorrhagic *E. coli* (EHEC) originated from EPEC that has additionally acquired *stx* genes via horizontal gene transfer (HGT) (10), enabling the secretion of Shiga toxins and efficient attachment to the host’s colonocytes (3). The resulting systemic consequences may even result in the death of infected people (6, 7, 11).

Foodborne diseases, including those caused by diarrheagenic *E. coli,* have particularly severe impacts on populations in low- and middle-income countries (LMICs) (12) due to restricted access to safe drinking water (13), poor healthcare provision (14), and low coverage of adequate sewage treatment (15, 16). Additionally, certain climate change impacts (e.g., rising temperature, heavy rainfall, and flooding) were associated with increased incidence of diarrheal diseases (17), highlighting the need for strategies to improve control over these illnesses. The establishment of robust diagnosis and systematic surveillance is a key measure to controlling the growing challenge of diarrheal diseases, whereby access to diagnostic tools is essential to improved treatment strategies (18) and better health outcomes (19).

Diarrheagenic *E. coli* is typically diagnosed in stool samples from diarrheagenic patients (20). For the assessment of community-level epidemiology, it is also important to diagnose pathogens in samples representative of the disease chain transmission, including asymptomatic patients. Wastewater-based surveillance has been acknowledged in that regard (21) and successfully implemented for COVID-19 surveillance in high-income areas (22). However, there is limited knowledge in implementing wastewater-based surveillance without sewer infrastructure, while half of the world’s population is now served by sanitation with on-site containments (16). As the fecal-oral route plays an important role in water- or food-borne *E. coli* transmission (20), wastewater from on-site containments could provide a basis for strategic monitoring and investigation of epidemiological spread in non-sewered areas (23).

The use of diagnostic tools such as polymerase chain reaction (PCR)-based approaches often complicates the establishment of robust surveillance in regions with limited access to expensive laboratory infrastructure like thermocyclers (24). Hence, there is a demand for the development of alternatives that are easy to use, independent of costly equipment, and field-applicable (18).

Loop-mediated isothermal amplification (LAMP) has gained attention as an alternative method for its independence of thermocyclers (25). It was first described by Notomi and colleagues in 2000 and operates at a constant temperature and with the use of at least four distinct primers. The amplification process is initiated by the annealing of the forward and backward inner primers (FIP and BIP) to the target strand, followed by elongation. Polymerases in LAMP must have strand displacement activity as the annealing of the forward and backward outer primers (F3 and B3) initiates the displacement of the previous elongation product. The resulting displaced single-stranded sequences are stabilized by the formation of loop structures at their ends, enabling them to serve as template sequences themselves (26). On top of that, they offer an additional binding site for forward and backward loop primers (LF and LB), which were introduced in later LAMP applications and accelerate the reaction (27). In addition to its independence of thermocyclers, LAMP demonstrated robust performance despite the presence of biological inhibitors (28). LAMP can thus be considered for the development of diarrheagenic *E. coli* detection assays.

LAMP assays targeting *eae* and *stx* present in EPEC, STEC, and EHEC have been developed in the past for food matrices (29–31), pure *E. coli* cultures (32), and fecal samples (31) and combined with different detection modes. The replication of nucleic acids in LAMP yields magnesium pyrophosphate (33) whose precipitation is recognizable with turbidity (29–31). However, given the high rate of false positives in LAMP (34) and noticeable spectrophotometric readings due to protein precipitation in clinical samples (28), some detection modes with improved specificity would be beneficial. Recently, the use of molecular beacons (MBs) with LAMP (MB-LAMP) provides a specific and exonuclease-independent approach (35). The formation of a hairpin structure of MB holds the fluorophore and quencher on each end in close proximity and only permits the emission of fluorescence upon hybridization to a LAMP amplicon (36).

Furthermore, the nucleic acid lateral flow (NALF) technology shows potential for specific pathogen diagnostics for infectious disease monitoring (37). Mukama and colleagues developed an NALF-based assay that allowed specific detection of LAMP amplicons using CRISPR-Cas cleavage, resulting in chromatic transformation on a lateral flow stripe (38). More simply, fluorophored primers, serving as probes, can be used to induce hybridization with immobilized complementary elements sitting on lateral flow devices, resulting in a visible band at the desired lateral flow strip line (39).

In this study, MB- and NALF-LAMP assays were developed for the specific detection of *eae* and *stx2* genes that are characteristic of the diarrheagenic *E. coli* types EPEC, STEC, and EHEC (3) and have been correlated to severe disease in the past (11, 40). Further, duplex LAMP assays for *eae* and *stx2* with two different detection modes were developed, and their sensitivities and specificities were evaluated and compared to the reference digital PCR (dPCR) assay. Finally, the assays were validated for performance within the environmental matrix of wastewater in on-site containments, relevant to epidemiological surveillance in non-sewered areas (16) and investigation of diarrheagenic *E. coli* -transmission pathways (20). Ultimately, reliable LAMP assays for the detection of *eae* and *stx2* in wastewater from on-site containments could support the systematic surveillance of foodborne EPEC, STEC, and EHEC, including in non-sewered areas.

## MATERIALS AND METHODS

### LAMP and PCR primers

LAMP primers for *eae* and *stx2* as previously published (31) were used with some modifications for two different detection modes: MB- and NALF-LAMP assays (Table 1). For MB, primer LF was expanded by six bases at the 5′ end that are complementary to the 3′ tail, allowing for the hairpin formation equipped with a fluorophore and a quencher at each end. For NALF, primers LF and LB were functionalized with biotin (or digoxigenin) and labeled with a fluorophore at the 5′ end. PCR primers for *eae* and stx2 from other studies (31, 41) were used for the dPCR assay, which was conducted to benchmark assay performance (Table S1).

**TABLE 1:** Sequences of primers for loop-mediated isothermal amplification (LAMP) assays for detection of *eae* and *stx2* with different detection modes, molecular beacon (MB), and nucleic acid lateral flow (NALF)^*a*^

Target	Primer		5′-Sequence-3′
*eae*	F3		TGACTAAAATGTCCCCGG
	B3		CGTTCCATAATGTTGTAACCAG
	FIP		GAAGCTGGCTACCGAGACTC CCAAAAGCAACATGACCGA
	BIP		GCGATCTCTGAACGGCGATT CCTGCAACTGTGACGAAG
	LF	MB	[TAMRA] TGACAA**GCCGCATAATTTAATGCCTTGTCA** [BHQ-2]
		NALF	[biotin] **GCCGCATAATTTAATGCCTTGTCA**
	LB	MB	**ACGCGAAAGATACCGCTCT**
		NALF	[FAM] **ACGCGAAAGATACCGCTCT**
*stx2*	F3		CGCTTCAGGCAGATACAGAG
	B3		CCCCCTGATGATGGCAATT
	FIP		TTCGCCCCCAGTTCAGAGTGA GTCAGGCACTGTCTGAAACT
	BIP		TGCTTCCGGAGTATCGGGGAG CAGTCCCCAGTATCGCTGA
	LF	MB	[FAM] GCTCCT**GCGTCATCGTATACACAGGAGC** [BHQ-1]
		NALF	[digoxigenin] **GCGTCATCGTATACACAGGAGC**
	LB	MB	**GATGGTGTCAGAGTGGGGAGAA**
		NALF	[FAM] **GATGGTGTCAGAGTGGGGAGAA**

^*a*^ For MB-LAMP, six bases were added to LF at 5’ for hairpin shape formation, and it was functionalized with a fluorophore, TAMRA and FAM for *eae* and *stx2*, and a quencher, BHQ-2, on each end. For NALF-LAMP, LF and LB were functionalized with biotin/digoxigenin and a fluorophore. Identical sequences of LF and LB for both detection modes are bolded.

LAMP and PCR primers were ordered from Microsynth (Balgach, Switzerland) in solid state and upon arrival rehydrated with nuclease-free water and stored at −20°C until use. The specificity of primers against targets was validated *in silico* by aligning them to the sequences for *eae* and *stx2* using the SnapGene software (Version 7.1.1; GSL Biotech, Illinois, USA) (Fig. S1).

### LAMP assays

All chemicals, including Tris-HCl, KCl, MgSO_4,_ and (NH_4_)SO_4_ for a buffered solution, 5 M betaine solution (Cat. No. B0300), and Tween 20 (Cat. No. P1379), were purchased from Sigma-Aldrich (St. Louis, MI, USA). *Bst* 3.0 DNA polymerase (Cat. No. M0374) and 10 mM dNTP solution mix (Vat. No. N0447) were purchased from New England Biolabs (Ipswich, MA, USA).

Per 25 µL of the LAMP reaction, 0.2 µM F3 and B3, 0.8 µM LF and LB, and 1.6 µM FIP and BIP were mixed with 1.4 mM dNTP, 8 U of the *Bst* polymerase, 0.8 µM betaine, and 0.1% Tween 20 in the buffered solutions of 20 mM Tris-HCl, 150 mM KCl, 8 mM MgSO_4,_ and 10 mM (NH_4_)SO_4_. Five microliters of the DNA template was added to the reaction. For a no-target control (NTC), 5 µL of nuclease-free water was added.

MB-LAMP reactions were run in a QuantStudio 3 (Applied Biosystems, Waltham, MA, USA) at 65°C for 60 minutes, with fluorescence recorded every minute. The data were analyzed in the QuantStudio Design software (Version 1.5.2, Applied Biosystems, Waltham, MA, USA) and Microsoft Excel (Microsoft Corporation, Redmond, WA, USA).

NALF-LAMP reactions were prepared in 0.2 mL PCR tubes and run in a heating block from a BentoLab Pro device (London, UK). The temperature was set at 65°C for 25 minutes, followed by a denaturation step at 90°C for 5 minutes. Ten microliters of the sample was pipetted on the HybriDetect 2T LF dipstick (Cat. No. MGHD2 1, Milenia Biotech GmbH, Giessen, Germany) and placed in a 1.5 mL Eppendorf tube with 100 µL of HybriDetect kit running buffer (Cat. No. MGHD2 1, Milenia Biotech GmbH, Giessen, Germany) (Fig. S2). After approximately 1 minute, the result became visible. Of note, the manufacturer of the HybriDetect 2T kit specified that the dipsticks were to be left in the buffer solution for a maximum of 15 minutes; (42) hence, the results were considered reliable within 1 to 15 minutes after placing the dipstick in the buffer solution.

All samples for MB-LAMP and NALF-LAMP assays were run in multiple replicates. For lower concentrations, typically more replicates were run due to their high variability.

### dPCR assays

For dPCR, Stilla Naica multiplex PCR mix 10X was purchased from Stilla Technologies (Cat. No. R10105, Villejuif, France), which consists of PCR buffers A and B. Per 25 µL of the reaction, 500 nM forward and reverse primers and 200 nM probe were mixed with PCR buffers A and B following the manufacturer’s instruction, 0.05 µM of fluorescein sodium salt (Cat. No. 0681, VWR, Radnor, PA, USA), and 5 µL of DNA template. The samples were loaded into Sapphire chips (Stilla Technologies, Villejuif, France), and the chips were transferred to the Geode (Stilla Technologies, Villejuif, France) for droplet generation and PCR. In the Geode, a partition phase at 40°C for 12 minutes preceded thermocycling, consisting of 45 cycles of 10 seconds at 95°C and 1 minute at 59°C. After the reaction, the chips were read and scanned in the Naica Prism3 using Crystal Reader (Stilla Technologies, Villejuif, France). The data were analyzed in CrystalMiner (Version 2.3.0, Stilla Technologies, Villejuif, France). All samples were run in duplicate.

### Sample preparation

**Fig 1:**
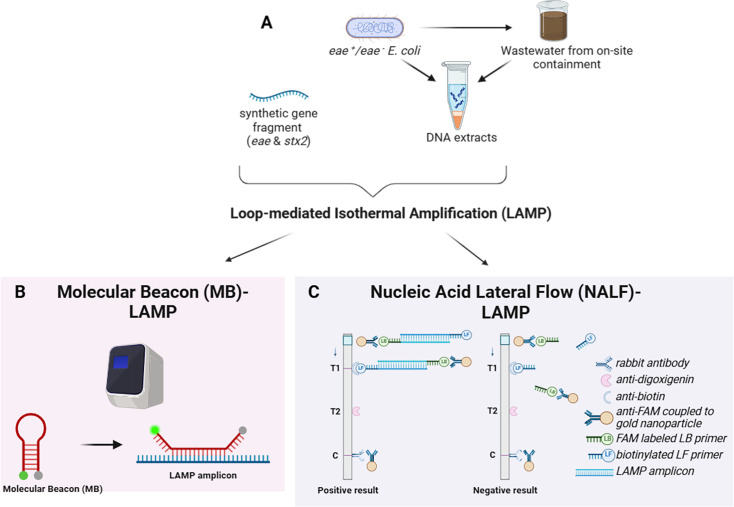
(A) Loop-mediated isothermal amplification (LAMP) assays were developed for the diarrheagenic *E. coli*-related *eae* and* stx2* genes. (B) A fluorescent molecular beacon (MB) was used for monitoring real-time amplification and quantification. (C) A nucleic acid lateral flow (NALF) detection was used as a point-of-care platform.

Synthetic gene fragments for *eae* and *stx2* served as targets in the initial development of the assays (Fig. 1A). Synthetic gene fragments were defined as the region spanned by all primers with an extension of 100 bp up- and downstream and ordered as solid state from Integrated DNA Technology (IDT; Coralville, USA). They were rehydrated upon arrival and stored at a stock concentration of 10^6^ copies per μL at −80°C until use.

Suspensions of *eae-*positive and -negative *E. coli* strains were used for assay validation (Fig. 1A). Two strains were isolated from wastewater, and the presence of *eae* was confirmed by whole-genome sequencing (43) and dPCR assays. Five hundred microliters of a cryogenic *E. coli* stock in 12% glycerol was cultured in 4.5 mL of lysogeny broth (LB) at 37°C for 6 hours with a shaking speed of 40 rpm. Afterward, the suspension was washed by centrifuging the culture tube at 3,000 g for 15 min, discarding the supernatant, and replacing it with phosphate-buffered saline (PBS) (1X) (Cat. No. AM9625, Thermo-Fisher, Waltham, MA, USA) three times. The colony-forming unit (CFU) was determined by plating on chromogenic agar (Cat. No. 90924, Sigma-Aldrich, St. Louis, MI, USA) in duplicates.

Additionally, wastewater samples spiked with suspensions of different *E. coli* strains were used for further validation (Fig. 1A). A wastewater sample was collected from the supernatant layer on an on-site containment from a household in Kampala, Uganda, in January 2023, within the scope of another project. Ten microliters of selected *E. coli* was mixed with 990 µL of wastewater and incubated for 20 minutes with a shaking speed of 220 rpm at room temperature.

DNA from *E. coli* strain suspensions was extracted using the QIAamp Fast DNA Stool Mini Kit (Cat. No. 51604, Qiagen, Hilden, Germany) following the manufacturer’s protocol (44). Briefly, 0.2 mL of a bacterial suspension in PBS (1X) was taken as a starting volume for the extraction protocol. The eluted DNA (extract with a volume of 200 µL) was further purified using the OneStep PCR Inhibitor Removal Kit (Cat. No. D6030, Zymo Research, Irvine, CA, USA). DNA extracts from wastewater samples showing no naturally occurring *eae* and *stx2* genes, as confirmed by dPCR assays, were used as blank controls.

Due to biosafety constraints of the *stx2*-positive *E. coli* strain, the synthetic *stx2* fragment was spiked into a blank control to validate the assays in wastewater matrices.

For the specificity test, wastewater samples spiked with the *eae*-negative and -positive strains underwent the aforementioned DNA extraction. DNA extracts were then mixed with selected dilutions of synthetic *stx2* fragments. High specificity was defined as the ability of the assay to distinguish well between those samples that are reflective of EPEC (*eae-*positive, *stx2-*negative), STEC (*eae-*negative, *stx2-*positive), EHEC (*eae-*positive, *stx2-*positive), and other non-diarrheagenic *E. coli* (*eae-*negative, *stx2-*negative).

All DNA extracts from wastewater samples were diluted threefold for dPCR or LAMP assays to minimize impacts of co-purified inhibitory substances.

### Data analysis

The concentrations of *eae* and *stx2* from synthetic gene fragments, and DNA extracts were calculated by absolute quantification of the dPCR assays. The calculated concentrations were within the magnitude of the manufacturer’s estimated concentrations.

For MB-LAMP(Fig. 1B), the change in the fluorescence signal, ∆RN, was monitored with reaction time. The time to reach the fluorescence threshold (T_t_) was calculated with different concentrations of targets. The T_t_ and logarithmic concentration were plotted, and the best-fit regression line with an R^2^ between the two was derived to assess the quantitative capability. The limit of detection (LoD) in the MB-LAMP assays was defined as a concentration of synthetic gene fragments that was significantly distinguishable from the NTCs. *t*-tests in RStudio (Version 2023.9.1.494) were used to identify the target concentration, of which T_t_ differed from those of the NTCs on a significance level of 5%.

For NALF-LAMP, three lines, Test 1 and 2 (T1 and T2) and Control (C), were embedded in the dipstick (Fig. 1C). The results were considered valid when a red line was shown in C. The presence of *eae* and *stx2* was diagnosed by the red lines in T1 and T2. The LoD was identified as the target concentration, where 90% of all replicates were positive, making them well distinguishable from the NTCs.

## RESULTS AND DISCUSSION

### MB-LAMP assays

**Fig 2:**
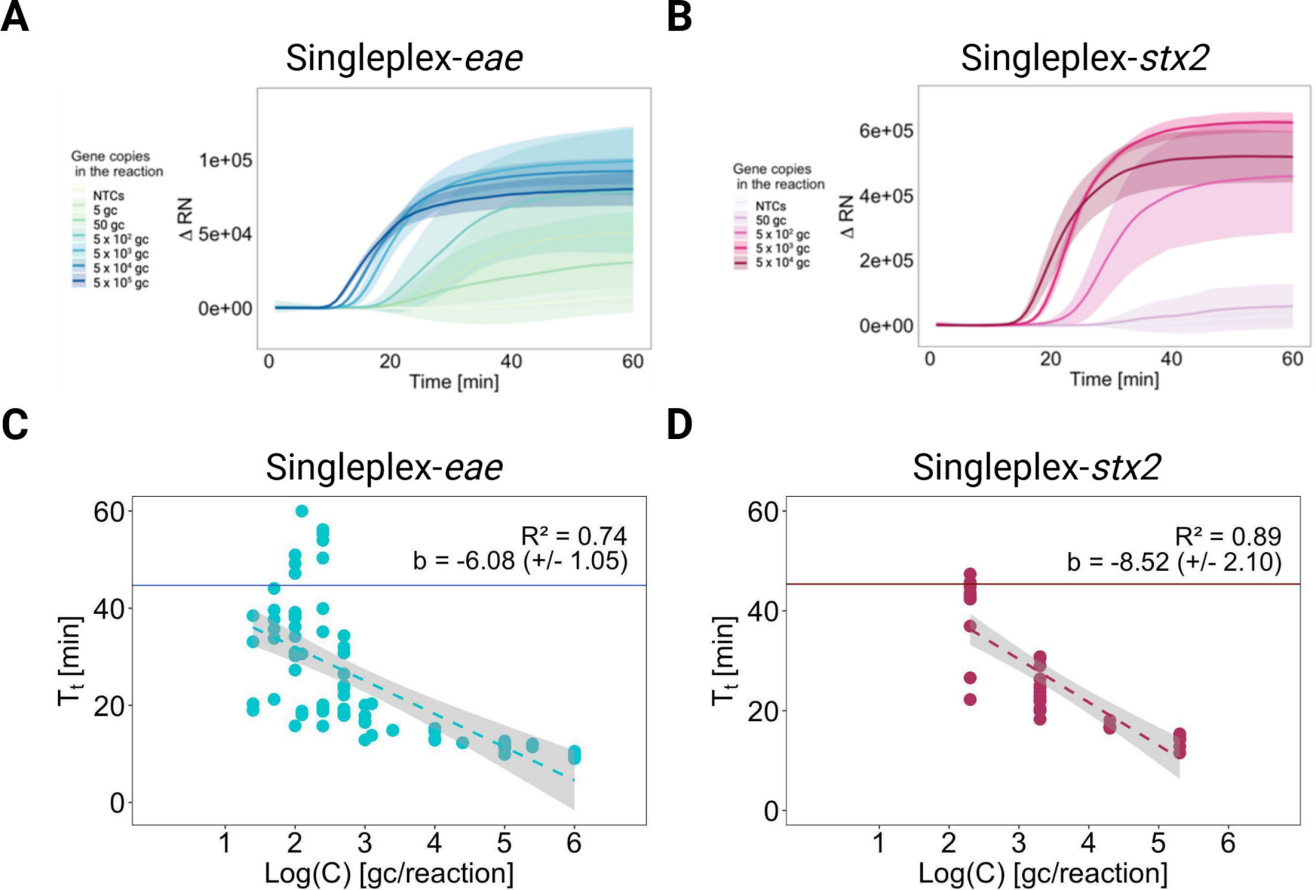
Quality assessment of two single-plex loop-mediated isothermal amplification (LAMP) assays with the use of molecular beacons (MBs) as fluorescent reporter molecules. (**A and B**) The real-time monitoring of change in fluorescence signals, ΔRN, in MB-LAMP reactions for detection of *eae* and *stx2*. The intensity of the color reflects the concentration of synthetic gene fragments estimated by digital PCR assay. No-target controls (NTCs) were run in parallel. (**C and D**) The plot between time to reach threshold (T_t_) and the logarithmic concentration of synthetic gene fragments. Dots indicate sample points. The dotted line and gray area indicate the best-fit regression line and 95% confidence region. The R^2^ value and slope of the regression line are shown. The horizontal lines indicate the average of T_t_ for NTCs.

Two singleplex MB-LAMP assays for *eae* and *stx2* were first developed in this study. During the LAMP reaction, the hairpin shape of MB is linearized by hybridization to LAMP amplicons. This leads to spatial separation of the fluorophore and quencher, thus resulting in an increased fluorescence signal (36) (Fig. 1B). Different concentrations of synthetic fragments induced different onset times of fluorescence signal increases. Accordingly, the fluorescence signal increased earlier with the higher target concentrations in the reaction (Fig. 2A and B). For the MB-LAMP assay with synthetic *eae* fragments, T_t_ with the concentration of ~10^6^ gene copies per reaction was 9.8 (Standard Deviation (SD) 0.5, *n* = 4) minutes, while a target concentration of ~10^1^ gene copies per reaction resulted in 32.3 (SD 8.5, *n* = 10) minutes (Fig. 2C). The MB-LAMP assay with synthetic *stx2* fragments showed similar concentration-dependent signal increases T_t,_ with the concentration of ~10^5^ gene copies per reaction being 13.9 (SD 1.2, *n* = 7) minutes, while a target concentration of ~10^2^ gene copies per reaction resulted in 40.0 (SD 7.8, *n* = 11) minutes (Fig. 2D). For both MB-LAMP assays, the T_t_ crossed the threshold after ~40 minutes in the NTCs, suggesting non-specific amplification toward the end of the reaction time. The assays had T_t_ of 44.7 (SD 9.1, *n* = 27) minutes for *eae* and 45.4 (SD 8.8, *n* = 15) minutes for *stx2* in NTCs. Concentrations at < ~10^2^ gene copies per reaction yielded amplification curves with large variability whose standard deviations (shaded areas in Fig. 2A and B) revealed overlap with those of NTCs. Consequently, they could not be reliably distinguished from NTCs.

To investigate the quantitative capability of the developed assays, T_t_ was plotted as a function of the log_10_ target concentration (Fig. 2C and D). This illustrated the degree of dependency of T_t_ on the target concentration (slope b), the accuracy of the data’s fit to a log-linear model (R^2^), and consequently, to what extent concentration is linearly dependent on T_t_ (quantitative capability). Both assays showed moderate log-linear relationships between the concentration and T_t_ with R^2^ of the best-fit lines of 0.74 and 0.89 for *eae* and *stx2* single-plex assays, respectively. The moderate relationship may be attributable to the greater variability of T_t_ with target concentrations below 10^3^ gene copies per reaction (Fig. S3). The inclusion of data points with low concentrations showing high variability contributed to modest linearity. The LoDs were defined at 500 gene copies per reaction for *eae* and 2,000 gene copies per reaction for *stx2* MB-LAMP assay. Thus, the LODs were in the range of a few hundred up to 2,000 gene copies per reaction as corresponding T_t_ were significantly different from the T_t_ for NTCs on a significance level of 5% (Fig. S4).

**Fig 3:**
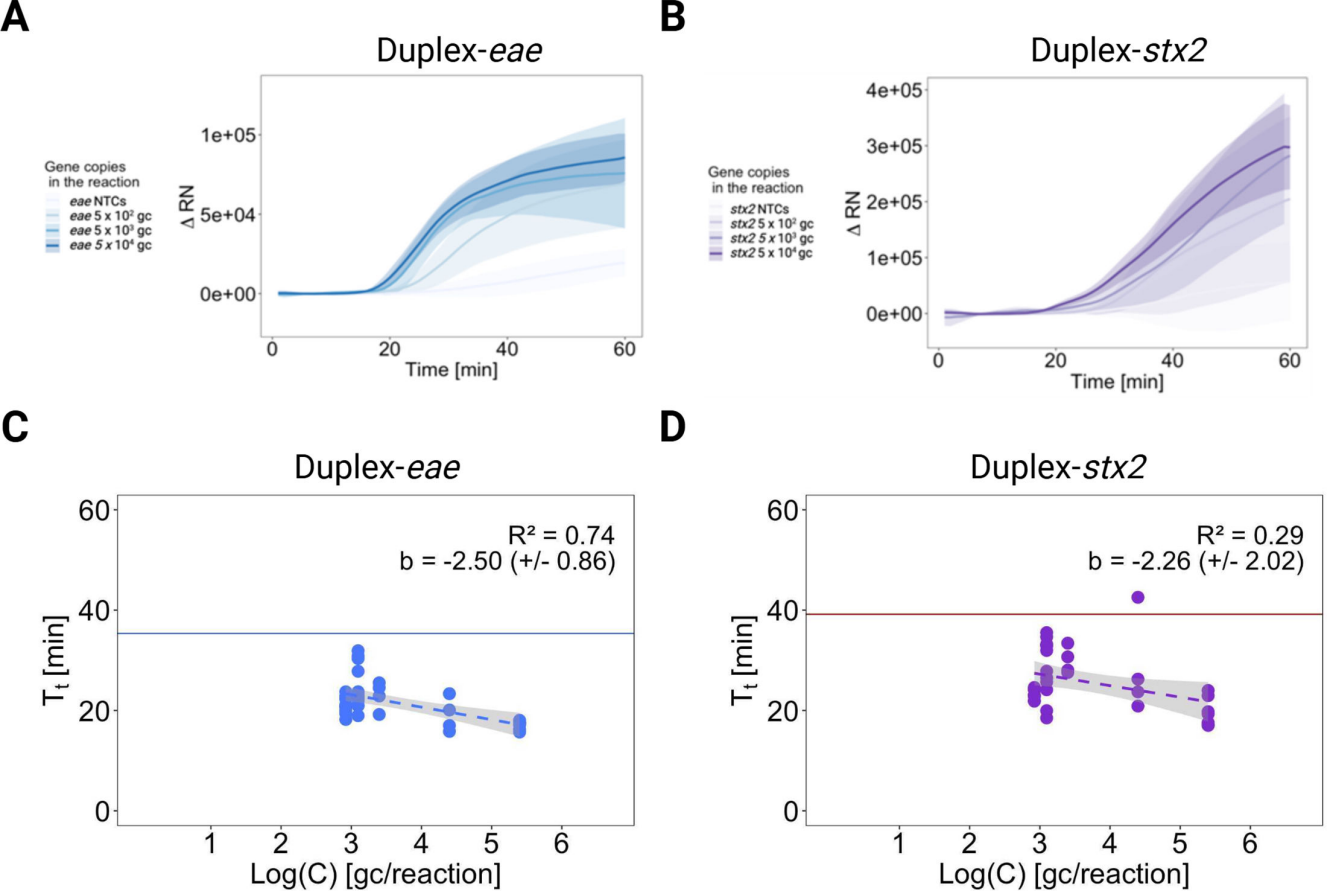
Duplex loop-mediated isothermal amplification (LAMP) assays with the use of molecular beacons (MBs) as fluorescent reporter molecules. (**A and B**) The real-time monitoring of change in fluorescence signals, ΔRN, in MB-LAMP reactions for detection of *eae* and *stx2*. The intensity of the color reflects the concentration of synthetic gene fragments estimated by digital PCR assay. No-target controls (NTCs) were run in parallel. (**C and D**) The plots between time to reach threshold (T_t_) and the logarithmic concentration of synthetic gene fragments. Dots indicate sample points. The dotted line and gray area indicate the best-fit regression line and 95% confidence region. The R^2^ values and slopes of the regression line are shown. The horizontal lines indicate the average of T_t_ for NTCs.

A duplex MB-LAMP assay for *eae* and *stx2* was also developed. For *eae*, T_t_ for the concentration of ~10^5^ gene copies per reaction was 17.1 (SD 0.8, *n* = 7) minutes, while a target concentration of ~10^3^ gene copies per reaction resulted in 23.1 (SD 3.6, *n* = 4) minutes. A T_t_ of 35.4 (SD 7.8, *n* = 14) minutes was determined for NTCs (Fig. 3C). For *stx2*, a concentration of ~10^5^ gene copies per reaction resulted in a T_t_ of 19.7 (SD 2.6, *n* = 7) minutes, while the T_t_ for a target concentration of 10^3^ gene copies per reaction was 26.9 (SD 4.8, *n* = 4) minutes. The T_t_ for NTCs was 39.2 (SD 15.7, *n* = 14) minutes (Fig. 3D). The linearity of the best-fit regression line was weaker in duplex detection with b = −2.50 (SD 0.86) and −2.26 (SD 2.02) minutes per log_10_ gene copies in the reaction for *eae* and *stx2* (Fig. 3C and D) than in single-plex detection with b = −6.08 (SD 1.05) and −8.52 (SD 2.10) minutes per log_10_ gene copies in the reaction, respectively (Fig. 2C and D). However, the large standard deviations of the slopes and the moderate R^2^ values, 0.74 and 0.29 for *eae* and *stx2* in duplex detection, indicate that the log-linear relationships observed are difficult to compare. Furthermore, the NTCs generally demonstrated earlier amplification in duplex compared to single-plex assays, illustrated by their smaller T_t_, 35.4 (SD 7.8, *n* = 14) and 39.2 (SD 15.7, *n* = 14) minutes for *eae* and *stx2* in duplex than ones in single-plex, 44.7 (SD 9.1, *n* = 27) and 45.4 (SD 8.8, *n* = 15) minutes for *eae* and *stx2*. The weaker linearity and earlier amplification of NTCs observed for the duplex indicate that a distinction between low concentrations and NTCs becomes more difficult than in singleplex. This is reflected in the slightly higher concentrations that were identified as LoDs in duplex compared to singleplex, based on the *t*-test in RStudio (confidence level = 95%, α = 5%). For the duplex, the LoD was calculated to range up to a few thousand gene copies per reaction, whereas for singleplex, the LoD lay within a few hundred to a few thousand gene copies per reaction. T_t_ (Fig. S4). Additionally, the amplification curves from concentrations below the respective LoDs (Fig. 2A, B and 3A, B) started to blend in with the mean of the NTCs.

**Fig 4:**
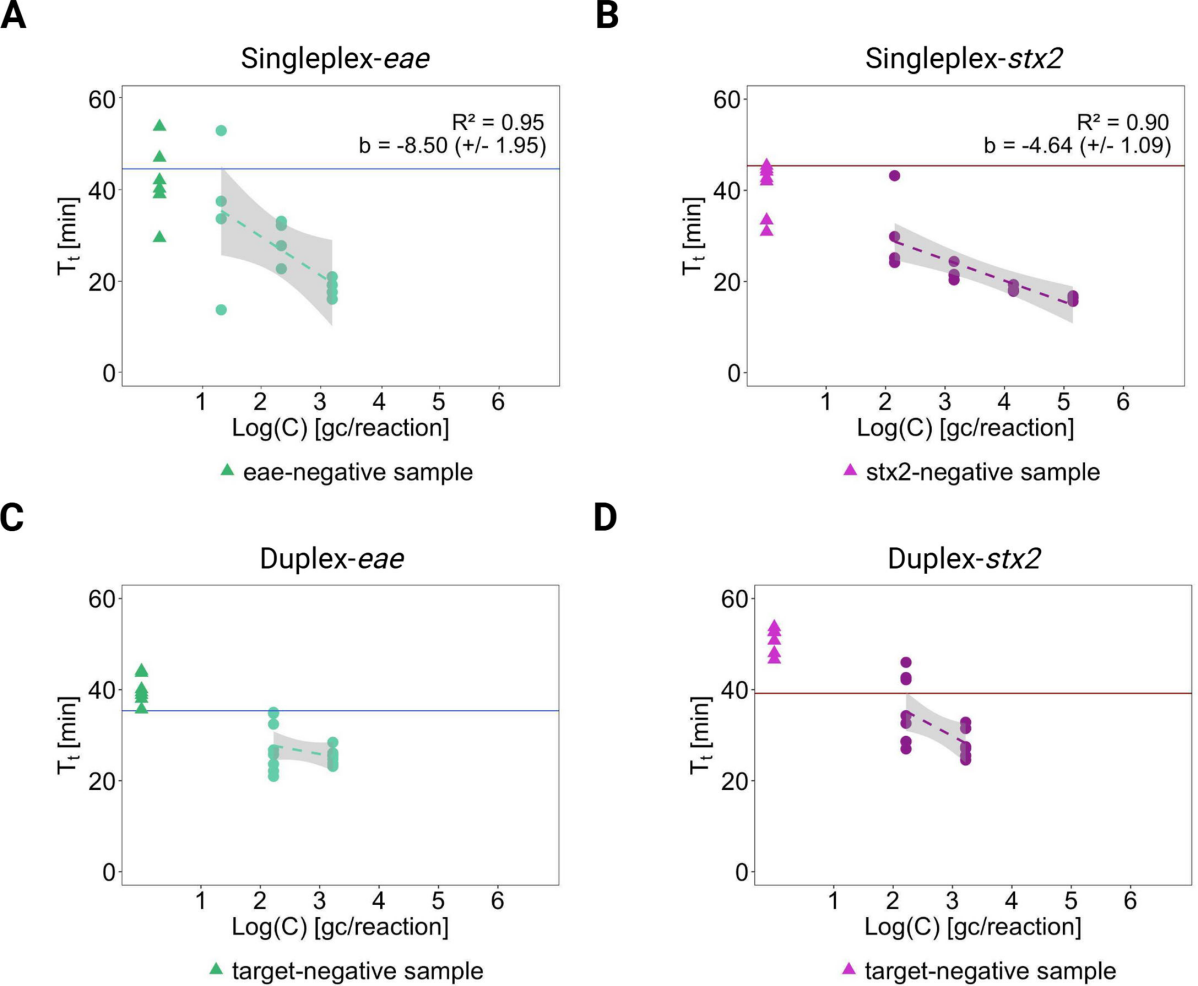
Validation of molecular beacon-based loop-mediated isothermal amplification (MB-LAMP) assays for detection of *eae* and *stx2* against environmental samples and wastewater from on-site containment. The plots between time to reach threshold (T_t_) and the logarithmic concentration of *eae*/*stx2* in wastewater matrices. Dots indicate sample points with *eae-*/*stx2-*spiked wastewater. Triangles indicate the sample points with *eae-*/*stx2-*negative wastewater, confirmed by digital PCR assay. The dotted line and gray area indicate the best-fit regression line and 95% confidence region. The horizontal lines indicate the average of T_t_ for NTCs. (**A and B**) Results of the singleplex MB-LAMP assays for detection of *eae* and *stx2*. The R^2^ values and slopes of the regression line are shown. (**C and D**) Results of the duplex MB-LAMP assay for detection of *eae* and *stx2*.

To investigate the performance of the developed assays in conditions realistic to non-sewered areas, the MB-LAMP assays were validated against wastewater matrices (Fig. 4). The results showed that both targets could be successfully detected in wastewater from an on-site containment, both with singleplex and duplex assays. However, the linearity between T_t_ and the logarithmic concentrations was less pronounced in wastewater (Fig. 4B) compared to synthetic *stx2* fragments in nuclease-free water (Fig. 2D). This was reflected in the slopes of their best-fit regression lines. The slope (coefficient b) of the singleplex detection of *stx2* in wastewater was −4.64 (SD 1.09) minutes per log_10_ gene copies in the reaction (Fig. 4B) whereas singleplex detection of synthetic *stx2* fragments in nuclease-free water (Fig. 2D) revealed a slope of −8.52 (SD 2.10) minutes per log_10_ gene copies in the reaction. For *eae* singleplex detection, no loss in linearity was observed in the wastewater matrix, b = −8.50 (SD 1.95) minutes per log_10_ gene copies in the reaction (Fig. 4A), compared to synthetic *eae* fragments in nuclease-free water, b = −6.08 (SD 1.05) minutes per log_10_ gene copies in the reaction (Fig. 2C). However, based on the large standard deviation of these coefficients and modest R^2^ for most best-fit regressions, the effect of the wastewater matrix on the log-linearity between T_t_ and target concentration in the reaction was not significant.

The purpose of the developed LAMP assays in this study was to distinguish diarrheagenic *E. coli* among EPEC, STEC, EHEC, and non-pathogenic *E. coli* that do not contain either of the targets in their genome. Therefore, DNA extracts from wastewater spiked with *eae*-negative *E. coli* suspensions were analyzed in both LAMP assays. The absence of *eae* and *stx2* in those samples was confirmed by dPCR analysis. These *eae*- and *stx2*-negative wastewater extracts were used as blank controls and yielded T_t_ close to those of positive wastewater samples with target concentrations around 10^2^ gene copies per reaction and lower (Fig. 4A and B). For the singleplex detection of synthetic *stx2* in wastewater extracts, for example, T_t_ of the blank controls was 40.5 (SD 5.4, *n* = 7), while those of wastewater extracts with 10^2^ gene copies per reaction were 30.6 (SD 7.6, *n* = 4). Thus, the difference in T_t_ for blank controls and wastewater extracts with low target concentrations remained non-significant. A high variability in T_t_ and similar data point distributions for wastewater extracts with low target concentrations and blank controls was observed in the duplex MB-LAMP assay (Fig. 4C and D). In the duplex MB-LAMP assay, *eae* detection in blank controls resulted in T_t_ of 40.0 (SD 2.8, *n* = 7), while those of wastewater extracts with 10^2^ gene copies per reaction were 27.7 (SD 5.3, *n* = 8). With the availability of the real-time amplification data, however (Fig. S5), it became apparent that, despite reaching the fluorescent threshold, the change in fluorescence, ∆RN, in blank controls remained very small, while wastewater extracts containing the targets increased in fluorescence. Hence, for reliable differentiation between blank controls and wastewater extracts with 10^2^ gene copies per reaction and lower, real-time amplification data improved judgment compared to differentiation solely based on T_t_.

### NALF-LAMP assays

**Fig 5:**
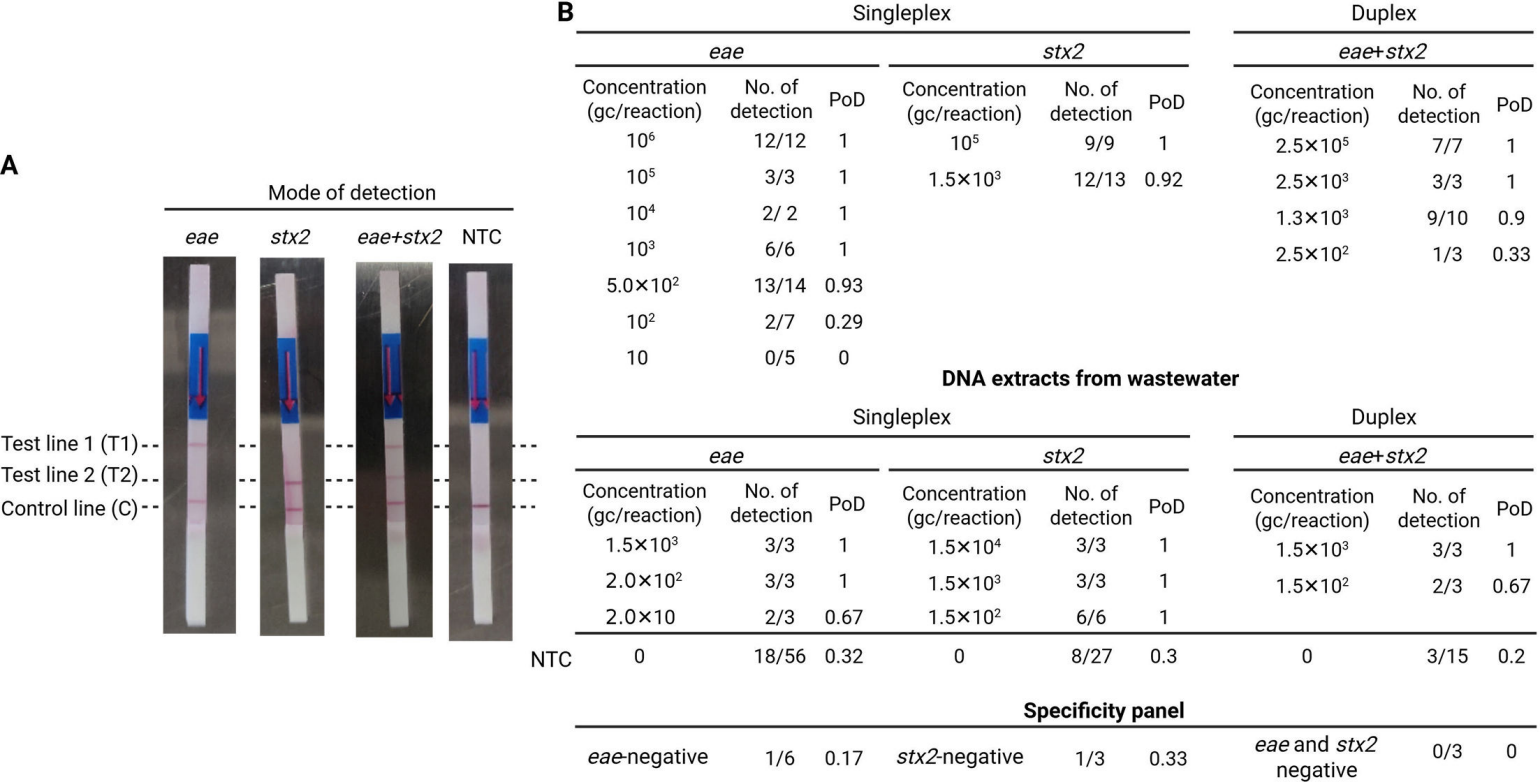
Nucleic acid lateral flow-based loop-mediated isothermal amplification (NALF-LAMP) assays for detection of *eae* and *stx*2. The positive result of targets was indicated by visible test lines, T1 and T2 for *eae* and *stx2,* respectively. The probability of detection (PoD) was calculated by dividing the number of detected samples out of the number of replicated samples (**A**) Pictures of dipsticks with three test lines, T1, T2, and control with the examples of *eae*, *stx2*, *eae* and *stx2*, and no-target control (NTC), respectively. (**B**) Overview of results of NALF-LAMP assays for *eae* and *stx2* in singleplex and duplex modes. NALF-LAMP assays were future validated in the wastewater matrix, and specificity was also evaluated using *eae*- and *stx2*-negative wastewater extracts.

The lateral flow dipsticks used in this study worked via the hybridization of functionalized primers to complementary elements on the test stripe. Gold-labeled anti-FAM antibodies bind to the amplicons due to the FAM-labeled LB primer and move across the dipstick by capillary forces until they are captured by anti-biotin and anti-digoxigenin antibodies, resulting in stained test lines (Fig. 1C) (42). Primers were designed to target *eae* and *stx2* in T1 and T2 due to their respective labeling with biotin or digoxigenin. Positive test results were identified by one or two stained test lines for the singleplex and duplex mode, respectively (Fig. 5A).

Figure 5 provides an overview of the rate of detected samples out of the total number of replicates in NALF-LAMP (probability of detection = PoD) with different concentrations of *eae* or *stx2* in nuclease-free water and wastewater matrices. For singleplex *eae* assay, T1 was visible with high concentrations at ~10^6^ gene copies per reaction, yielding a PoD of 1 (95 % CI: [0.74, 1]), with 12/12 replicates producing positive test lines, whereas low concentrations at ~10^2^ gene copies per reaction were only detected in 2 out of 7 replicates, resulting in a PoD of 0.29 (95 % CI: [0.04, 0.71]). Similar results were observed for the singleplex *stx2*, where concentrations around 10^5^ gene copies per reaction generated stained test lines in all nine replicates (PoD = 1, 95% CI: [0.66, 1]), and 12/13 tests were positive for concentrations at ~10^3^ gene copies per reaction (PoD = 0.92, 95% CI: [0.64, 1]). In the duplex mode, PoD was 1 for concentrations at >2.5×10^3^ gene copies per reaction, and 9/10 replicates were positive for 1.3 × 10^3^ gene copies per reaction (PoD = 0.9, 95% CI: [0.56, 1]).

Based on these results, the LoD in NALF-LAMP was identified at the concentration where at least 90% of the replicates were positive (PoD ≥0.9), as indicated by two or three visible test lines for singleplex and duplex assays, respectively (Fig. 5A). A target concentration in the range of a few hundred to a few thousand gene copies per reaction was identified as the LoD in both singleplex and duplex modes (Fig. 5B), leading to 13/14, 12/13, and 9/10 positive samples for *eae*, *stx2*, and duplex mode, respectively.

Like the MB-LAMP assay, the performance of the NALF-LAMP assay was assessed against wastewater extracts (Fig. 5B). The concentrations at around LoDs in wastewater extracts were reliably detected; e.g., 2.0 and 1.5 × 10^2^ gene copies of *eae* and *stx2* per reaction showed a PoD of 1, 3/3 and 6/6 positive samples (95% CI of the PoD: [0.29, 1] for *eae* and [0.54, 1] for *stx2*). However, it should be noted that the detection rates of these results must be considered carefully as PoD and hence the false positivity rate in NTCs were 0.32 for *eae* (95% CI: [0.20, 0.46]), 0.3 for *stx2* (95% CI: [0.14, 0.5]), and 0.20 for the duplex assay (95% CI: [0.04, 0.48]).

Furthermore, a specificity test was conducted in NALF-LAMP with the same blank samples used to assess the specificity in the MB-LAMP assay. Similar to the false-positive rates in the NTCs (~0.3), some target-negative wastewater samples showed positive test lines (Fig. 5B). Consequently, it was defined that three out of three replicates must show a visible test line to consider it being positive for a target. The probability of a false-positive result with colored test lines in three dipsticks was (0.3) (3) = 2.7%, which is acceptably low. Following this rule, none of the blank controls in the wastewater samples would be considered detectable in NALF-LAMP anymore. Additionally, wastewater extracts with target concentrations lower than the LoD, a few hundred to a few thousand gene copies per reaction, were not detectable in triplicates and were thus considered undetectable.

### Sensitivity and specificity

Both MB- and NAFL-LAMP assays developed in this study showed moderate sensitivity and specificity toward *eae* and *stx2*. Given that previous literature claimed equivalent or superior sensitivity in LAMP platforms compared to PCR when targeting *E. coli*-related genes (29, 31, 45), the sensitivity of the LAMP assays developed here is not in agreement. The study that was used as a basis for LAMP primer design in this study (31) achieved detection limits that are 100-fold lower than the LAMP assays at hand, even though similar sensitivities would have been expected because identical primer sequences were used. Despite the previously demonstrated specificity (31) and success in targeted annealing *in silico* (Fig. S1), dimerization and self-priming may still have led to non-specific amplification. Self-priming amplification increases false-positive results, generating background signals that hamper the precise distinction between the detection of low target concentrations and NTCs. Previous research has identified secondary structures in gel electrophoretic analyses of LAMP amplicons that likely resulted from self-priming amplification (46). In addition, the manufacturer of the dipsticks for NALF-LAMP assay illustrates that the choice of labeled primers could affect the risk of dimerization, causing false positives (47). A previous LAMP application tried to overcome the issue of false-positive amplification by designing and testing ten different primer sets to subsequently choose the combination that did not cause false positives and thus culminated in the highest sensitivity (48). This is also believed to be an issue of the reaction conditions. Liu and colleagues (35), for instance, varied the reaction temperature as well as the MB sequence length and MB concentration to optimize their assays’ performance. Additionally, the implementation of a temperature gradient for optimized annealing of the primers could be considered. This has been done previously to improve the performance of PCR assays for *eae* and *stx* (49) and may also be applied in LAMP. Furthermore, the addition of pullulan to the reaction mixture could result in higher specificity, as suggested by the finding that pullulan led to a delay in non-specific amplification (50). This effect could be observed in a concentration-dependent manner and interdependence with the amount of *Bst* DNA polymerase, while the assays’ performance was not negatively impacted. It was mainly explained by the perfectly linear structure of pullulan, which hindered LAMP primers from forming dimers by improving their stability in the reaction solution. Another important limitation, particularly in MB-LAMP, that could explain part of the false positivity observed in NTCs based on T_t_ is the software’s inability to accurately calculate T_t_ and distinguish real fluorescence emission from background noise in some cases. This would explain why, only given T_t_, false positivity seems worse than when additionally considering real-time amplification that shows how low the increase in fluorescence in NTCs and blank controls remains. Moreover, the necessity to open reaction tubes after amplification and pipette the content on dipsticks for NALF detection may explain some of the false positivity observed as this process increases the risk of cross-contamination of the working environment. In this regard, MB-LAMP bears a smaller risk for false positives as reaction tubes remain fully closed throughout the amplification and detection process. This is a drawback of the otherwise very simplistic NALF technology and highlights the importance of strict adherence to good laboratory practice as well as the exploration of other methods to maintain a low rate of false positivity, as they were discussed above.

Another possible reason for the relatively poor sensitivity observed in the LAMP assays at hand could be explained by differences in detection methods compared to the reference literature. The study by Wang et al. (2012) that was used as a basis for LAMP primers (31) used turbidity as a method of detection, which could result in the underestimation of the LoD due to non-specific amplification in the samples. In another study by Liu et al. (2017) using targeted MB detection for the diagnosis of other pathogens, the sensitivity was in a similar range to the one identified here (35). Consequently, reference sensitivities derived from non-specific detection methods should be considered with care as the reported LoDs can be underestimated.

Finally, it is important to mention that the LoD defined in this study (a few hundred to a few thousand gene copies in the reaction for singleplex and duplex assays) is based on synthetic gene fragment concentrations that were manually prepared and calculated based on the measured concentration in dPCR of synthetic gene fragments solutions with lower dilution factors. These samples with higher synthetic gene fragment concentrations (10^4^ gene copies per reaction and above) had narrow variability in dPCR measurements and were thus reliable enough to derive the low concentrations (around the LoD) with the inclusion of the applied dilution factor. Hence, the assumed concentrations are not highly accurate, and the LoD at hand may be slightly over- or underestimated. Furthermore, as the manual preparation of low synthetic gene fragment concentrations (10^2^ gene copies per reaction and below) was difficult, it was sometimes not possible to run the LAMP assays with all desired concentrations. This is why the LoDs at hand should be understood as ranges of concentration. For *stx2* singleplex, for instance, the true LoD may likely fall in the range between 200 and 2,000 gene copies per reaction (Fig. S4). But no manually prepared synthetic *stx2* fragment dilution matched this range of concentration based on the calculations. Hence, the LoD was set at the lowest concentration that presented with significantly different T_t_ from the NTCs on a 5% level.

When taking the recovery rate of the DNA extraction protocol into account, both LAMP assays were able to yield reliable positive results for spiking levels of approximately 10^5^–10^6^ CFU/mL wastewater. This concentration corresponds to the shedding level found previously in fecal samples from individuals infected with EPEC (51). Considering the very low infectious dose of diarrheagenic *E. coli* (< 100 cells) (52) and that wastewater from an on-site containment is somewhat diluted, selective enrichment cultures, like they are applied for the diagnostic detection of single pathogenic cells in 25 g of food, may be necessary to diagnostically detect low cellular levels within a biological sample using LAMP (29, 31). Additionally, the more specific method of enrichment involving immunomagnetic bead separation of a target strain, followed by resuspension and incubation, could be considered (53).

### Conclusions and outlook

We developed LAMP assays for the detection of *eae* and *stx2* gene sequences. The amplification of these targets allows the detection and crude discrimination of various strains of diarrheagenic *E. coli*, such as EPEC, STEC, and EHEC. For the detection of the resulting amplicons, we applied two different detection modes, an MB-based fluorescence detection and an NALF-based colorimetric detection by eye. The assays were duplexed and validated against wastewater from on-site containments, demonstrating the potential for application of the assays to environmental monitoring in non-sewered areas. The genetic targets are characteristic of various strains of diarrheagenic *E. coli*, EPEC, STEC, and EHEC and allow for their detection. The key benefits of LAMP are time and cost efficiency. LAMP can amplify genes within an hour, and isothermal reaction does not require expensive thermocyclers. Thus, the developed assays in this study have great potential for use in resource-limited settings. Two detection modes have different strengths and limitations that can be options for different deployment settings. Real-time monitoring of fluorescence signals in MB-LAMP assays not only provides an in-depth temporal understanding of amplification with potential quantitative capability but also takes non-specific amplification into account effectively for analysis, which can hardly be avoided in LAMP. However, it still requires a fluorescence reader, which can be a hurdle in financially constrained settings. The main benefits of NALF-LAMP assays are low cost and simple read-out. The paper-based dipstick methodology enables instrument-free analysis of targets, which does not require any professional personnel to use. Consequently, the estimated costs of NALF-LAMP assays (1,370 USD for one experimental run = 11 samples) are much lower than for the other assay types. For MB-LAMP, the cost for the necessary device is about ~50,000 USD, and dPCR equipment with an incorporated fluorometer and thermocycler approximately costs 65,000 USD (Table S2).

## Data Availability

All data have been deposited in the Eawag Research Data Institutional Collection (ERIC) for public availability at https://doi.org/10.25678/000E9D.

## SUPPLEMENTAL MATERIAL

Supplemental material aem.00880-25-s0001.docx Tables S1 and S2; Fig. S1 to S5.

Graphical abstract aem.00880-25-s0002.tiff Visual diagram of the study.

ASM does not own the copyrights to Supplemental Material that may be linked to, or accessed through, an article. The authors have granted ASM a non-exclusive, world-wide license to publish the Supplemental Material files. Please contact the corresponding author directly for reuse.
