# Supplementary material for: Development of loop-mediated isothermal amplification (LAMP) assays for the detection of diarrheagenic E. coli in wastewater
Source: Appl Environ Microbiol. 2025 Aug 11;91(9):e00880-25. doi: 10.1128/aem.00880-25 (PMC12442385; doi:10.1128/aem.00880-25)
Supplement: Supplemental material — Tables S1 and S2; Fig. S1 to S5. [file aem.00880-25-s0001.docx]

| **Supplemental Material**  **Development of Loop-Mediated Isothermal Amplification (LAMP) Assays for the Detection of Diarrheagenic *E. coli* in Wastewater**  Meret Zimmermann^1,2^, Markus Schuppler^2^, Timothy R. Julian^1,3,4^, Seju Kang^1*^  ^1^Department of Environmental Microbiology, Eawag, Swiss Federal Institute of Aquatic Science and Technology, Dübendorf, Switzerland  ^2^Laboratory of Food Microbiology, Institute of Food, Nutrition and Health, Swiss Federal Institute of Technology Zurich, Zurich, Switzerland  ^3^Swiss Tropical and Public Health Institute, Allschwil, Switzerland  ^4^University of Basel, Basel, Switzerland  *Corresponding author: [seju.kang@eawag.ch](mailto:seju.kang@eawag.ch)  Postal address: Überlandstrasse 133, CH - 8600 Dübendorf  Keywords: *Loop-mediated isothermal amplification (LAMP), Diarrheagenic E. coli, Wastewater-based surveillance (WBS), Lateral flow, Point-of-care (POC)* | | | | |
| --- | --- | --- | --- | --- |
| **Target** | **Primer/Probe** | **5’-Sequence-3’** | | **Ref** |
| *eae* | Forward | CATTGATCAGGATTTTTCTGGTGATA |  | |
|  | Reverse | CTCATGCGGAAATAGCCGTTA |  |  |
|  | Probe | **HEX**-ATACTGGCGAGACTATTTCAA-**BHQ-1** |  |  |
| *stx2* | Forward | ATTAACCACACCCCACCG | ^29^ | |
|  | Reverse | GTCATGGAAACCGTTGTCAC |  |  |
|  | Probe | **FAM**-CAGTTATTTTGCTGTGGATATACGAGGGCTTG-**BHQ-1** |  |  |

**Table S1** Sequences of PCR primers and probe for *eae* and *stx2*

| ***eae*** |
| --- |
| **** |
| ***stx2*** |
| **** |

**Figure S1.** *In silico* specificity of the LAMP and PCR primers to the *eae* and *stx2* sequence

| 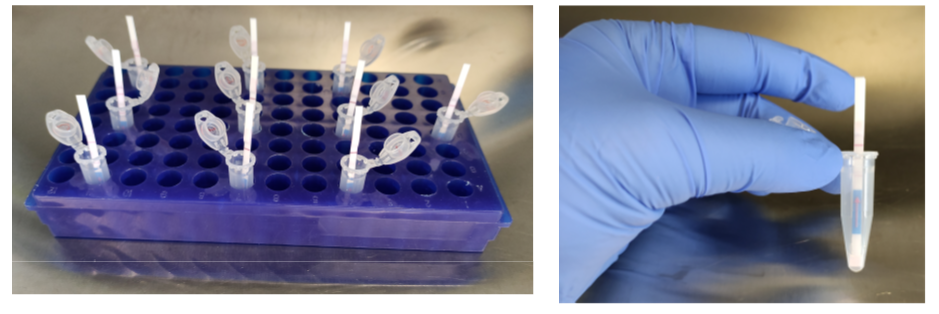 |  |
| --- | --- |

**Figure S2.** Set-up of the nucleic acid lateral flow (NALF) detection in loop-mediated isothermal amplification (LAMP) assays

The dipsticks used for NALF-detection following LAMP amplification only took a few minutes until read-out. A small volume (10 μL) of amplified sample was pipetted on the sample application area before placing the dipstick vertically in an Eppendorf tube holding 100 μL of running buffer supplied by the manufacturer. Capillary forces transported the sample and buffer upwards, where amplicons were captured on the test lines due to interactions between labeled loop primers annealed to them and complementary elements sitting on the dipstick.


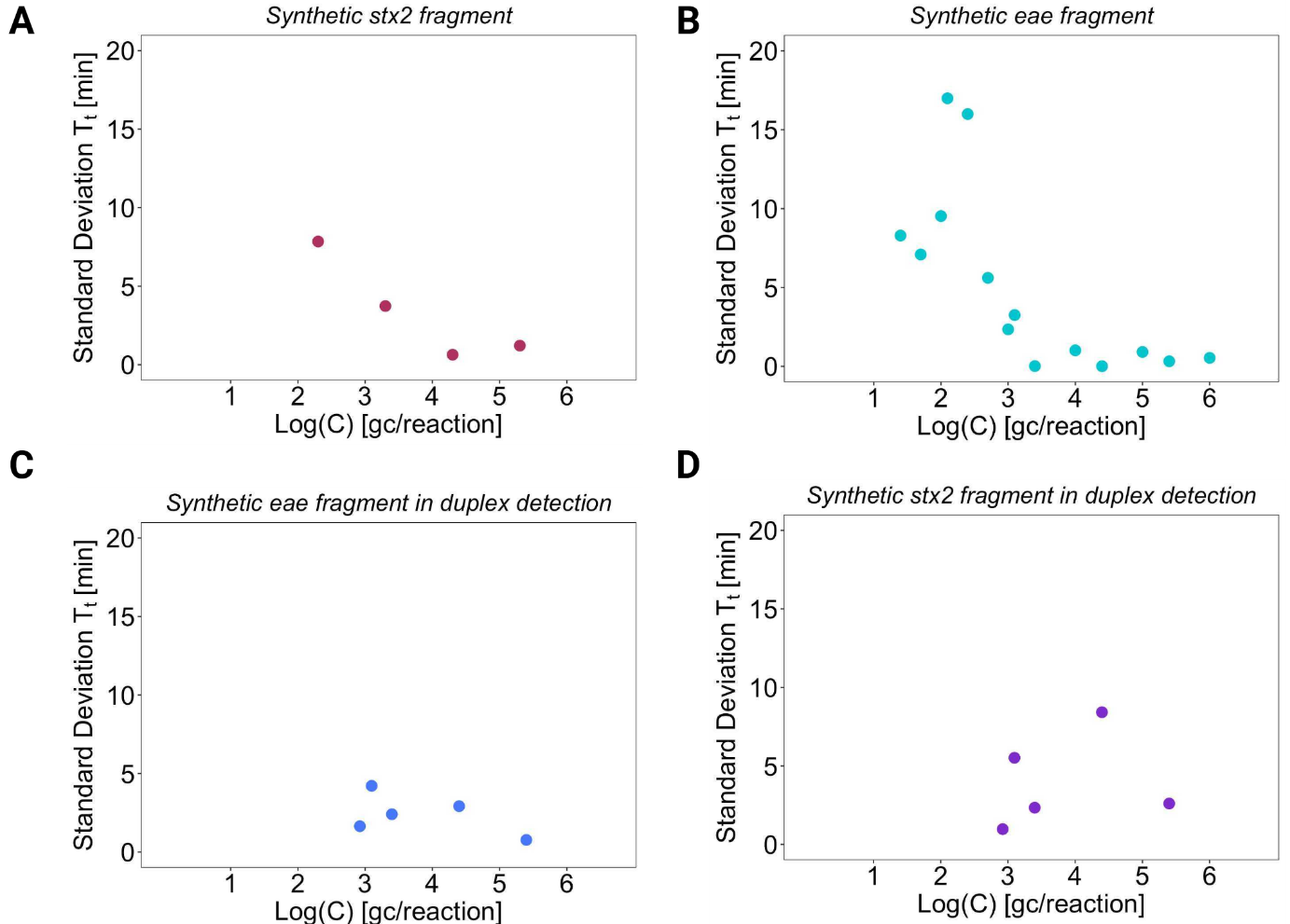


**Figure S3.** Standard deviation of T_t_ (time in minutes to reach the fluorescence threshold) with different logarithmic concentrations of *eae* and *stx2* for MB-LAMP assays (A and B) Two singleplex MB-LAMP assays for synthetic *eae* and *stx2* fragments (C and D) Duplex MB-LAMP assay for synthetic *eae* and *stx2* fragments.


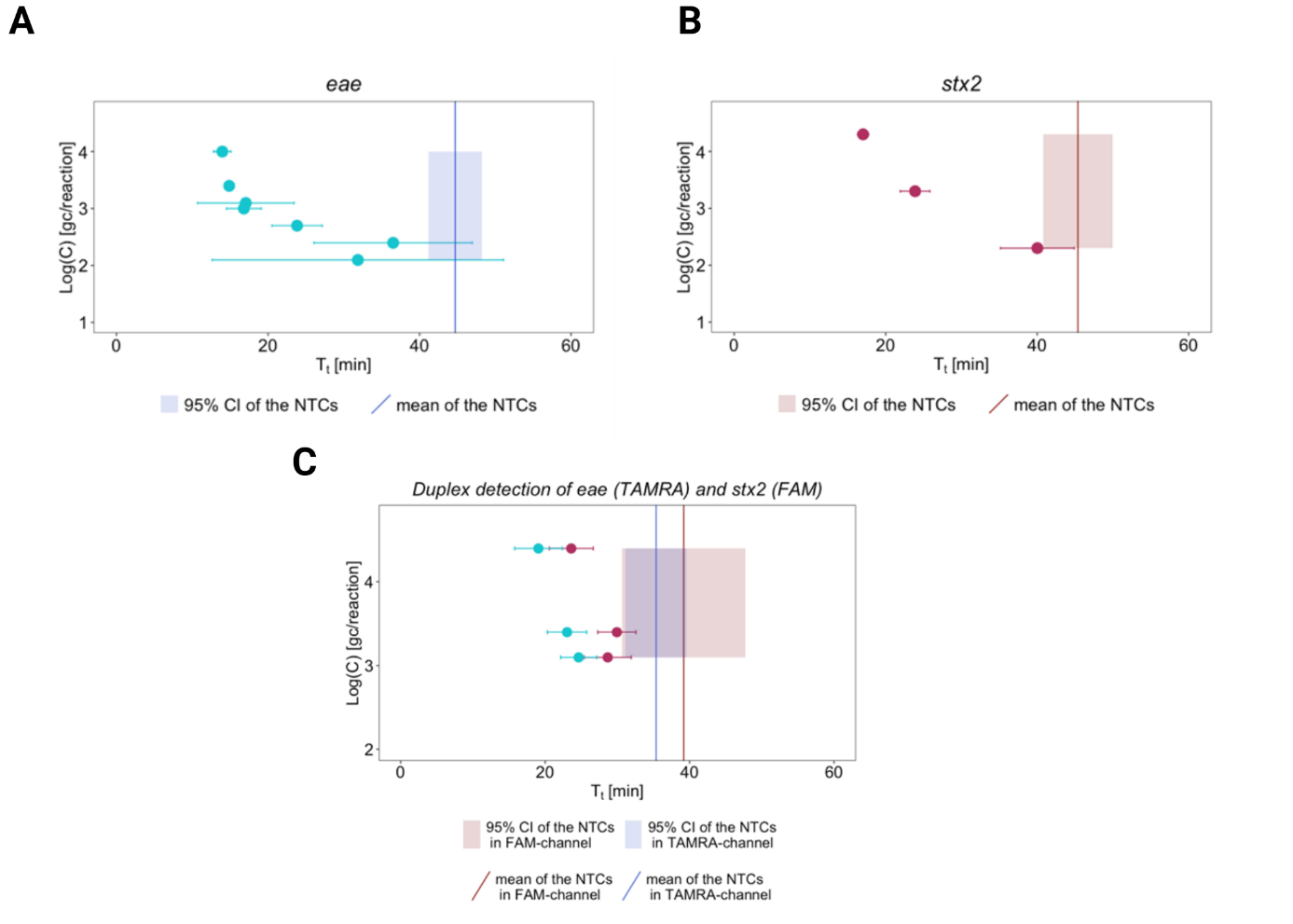


**Figure S4.** Graphical representation of the t-test conducted to define the Limit of Detection (LoD) in MB-LAMP assays at the synthetic *eae* or *stx2* fragment concentration that was significantly distinguishable from the no-target control (NTCs) based on their T_t_ (time in minutes to reach the fluorescence threshold).

| **Singleplex detection of *eae* and *stx2*** | | |
| --- | --- | --- |
| 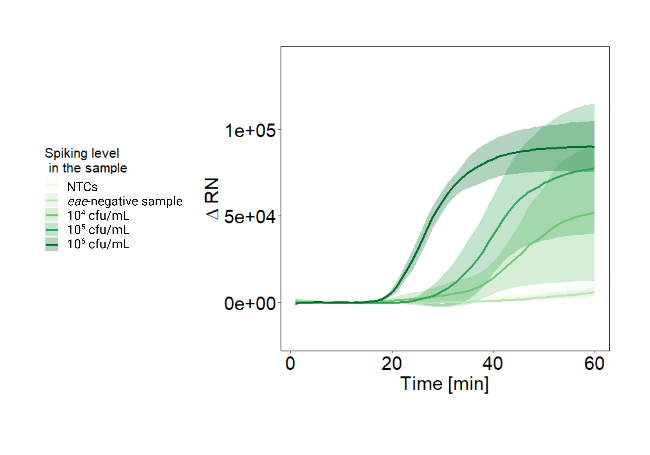 | | 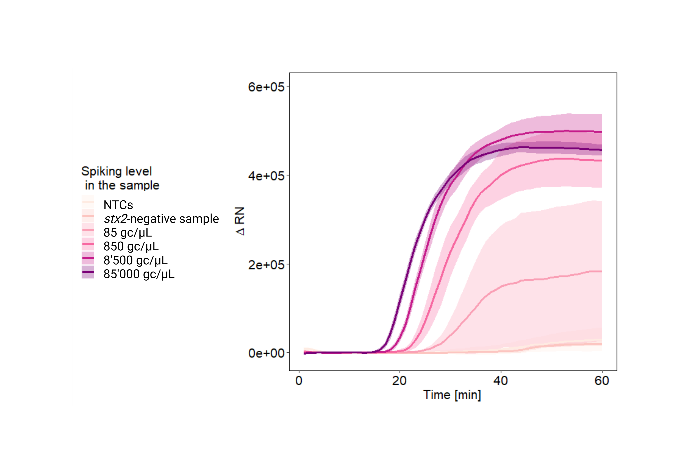 |
| **Duplex detection of *eae* and *stx2*** | | |
| **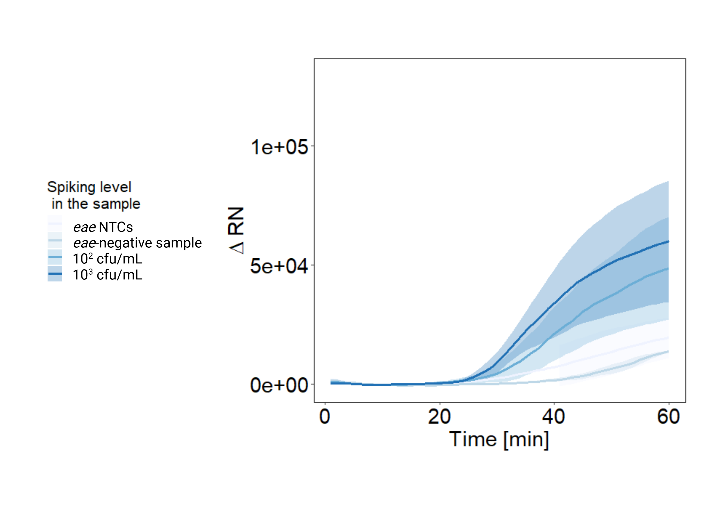** | **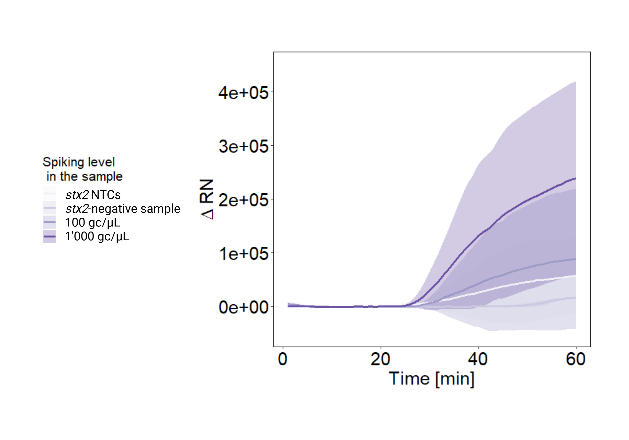** | |
|  | | |

**Figure S5.** Real-time amplification curves of molecular beacon (MB) detection coupled with LAMP assays (MB-LAMP) for *eae* and *stx2* in wastewater extracts (singleplex and duplex).

|  | **dPCR** | **MB-LAMP** | **NALF-LAMP** |
| --- | --- | --- | --- |
| **Reagents and Lab Consumables** | const. | const. | const. |
| **Equipment** | Stilla Sapphire Chips  = 100 USD | 1 MicroAmp 96-well plate = 13 USD  1 optical adhesive cover = 8.2 USD | 11 lateral flow dipsticks = 30 USD |
| **Devices** | Naica system  ~65’000 USD | QuantStudio3  ~50’000 USD | BentoLab = 1370 USD |

**Table S2.** Costs summarized for MB-LAMP, NALF-LAMP and digital PCR (dPCR). The costs for reagents and lab consumables were assumed to be constant as all assays required similar master mixes. The prices of the disposable equipment are calculated for 1 experimental run holding 11 samples.
