# Supplementary figures and images for: Development of loop-mediated isothermal amplification (LAMP) assays for the detection of diarrheagenic E. coli in wastewater
Source: Appl Environ Microbiol. 2025 Aug 11;91(9):e00880-25. doi: 10.1128/aem.00880-25 (PMC12442385; doi:10.1128/aem.00880-25)

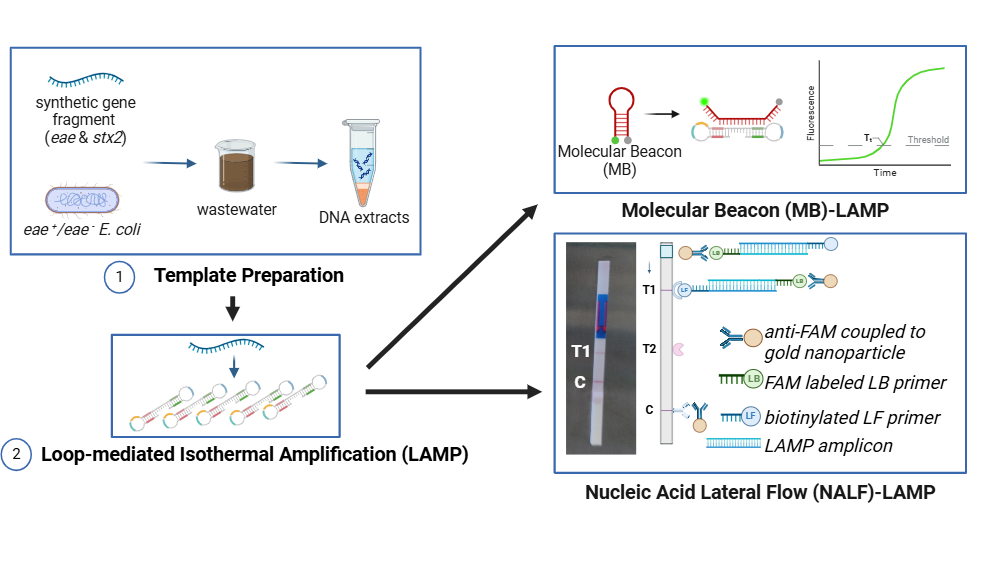

Supplement: Graphical abstract — Visual diagram of the study. [file aem.00880-25-s0002.tiff]
